# Neglected subluxation of the hip after trauma: A case report

**DOI:** 10.1016/j.amsu.2020.07.035

**Published:** 2020-07-22

**Authors:** Shohei Matsubayashi, Eri Kanzaki, Ritsu Tsujimoto, Makoto Osaki, Akifusa Wada

**Affiliations:** a Department of Orthopedic Surgery, Graduate School of Biomedical Sciences, Nagasaki University, Japan; b Department of Orthopaedic Surgery, Saga Handicapped Children's Hospital, Japan

**Keywords:** Limited motion, Shenton line, Hip subluxation, CT, Computed tomography, MRI, Magnetic resonance imaging, IPO, incomplete periacetabular osteotomy, ROM, range of motion

## Abstract

**Introduction:**

In children, the pelvis contains a large amount of cartilage components; therefore, when traumatic hip dislocation spontaneously reduces, it can be impossible to see on X-ray or computed tomography (CT) images in some cases, which can delay its detection.

**Case presentation:**

We report the case of a 10-year-old boy who was injured by being hit by a car while walking. Upon diagnosis of pelvic ring fracture, the patient received conservative treatment. Seven months after injury, the patient was referred to our department with the chief complaint of limping.

**Diagnosis:**

Marked limitation was observed in the left hip with extension of −40°, abduction of 10°, and internal rotation of 20°. X-ray revealed narrowing of the left hip joint space, with deformity of the femoral head, obturator foramen narrowing, and the break in the Shenton line. CT revealed proximal dislocation of the posterior acetabular wall and posterior subluxation of the femoral head. Magnetic resonance imaging (MRI) revealed necrosis of the femoral head.

**Intervention:**

Operation was performed with soft tissue dissection, varus-extension-internal rotation femoral osteotomy, greater trochanteric epiphysiodesis, and pelvic osteotomy (incomplete periacetabular osteotomy: IPO). After operation, complete paralysis of the sciatic nerve was observed.

**Outcomes:**

At 1 year after operation, the patient's limited range of motion (ROM) and femoral head necrosis had improved. The sciatic nerve paralysis had fully recovered.

**Conclusion:**

If hip extension, abduction, and internal rotation are limited and X-ray reveals a break in the Shenton line., subluxation of the hip should be suspected.

## Highlights

• Pelvic fractures in children.
• Limited ROM in hip.
• Break in Shenton line.
• Subluxation of hip.

## 1 Introduction

Traumatic hip dislocation in children is a rarely seen injury [1,2], and cases accompanied by acetabulum fracture are even more rare [3]. The majority of this injury is a consequence of high-energy trauma which includes motor vehicle accidents or falls from significant heights [3]. The symptoms are pain and inability to walk [1]. The typical deformities of posterior dislocation are flexion, adduction and internal rotation of the hip [1]. Good-quality X-ray evaluation is essential for confirming the diagnosis and revealing the type of dislocation [1]. CT's use is growing because it is the best modality to assess the sacrum, sacroiliac joints and acetabulum [2]. Neither CT scans nor X-ray studies are useful for assessing cartilage. MRI, on the other hand, is an important tool because it allows for assessment of the cartilage components of the immature pelvis [2]. In children, the pelvis contains a large amount of cartilage components [1]. Therefore, when traumatic hip dislocation spontaneously reduces, it can be impossible to see on X-ray or CT, which can delay detection. Here, we report our experience with a child with traumatic hip dislocation that spontaneously reduced, who gradually developed subluxation thereafter, which was discovered due to limping and limited range of motion (ROM) of the hip.

This study was conducted and reported in line with SCARE 2018 criteria [4].

## 2 Case presentation

**Fig. 1:**
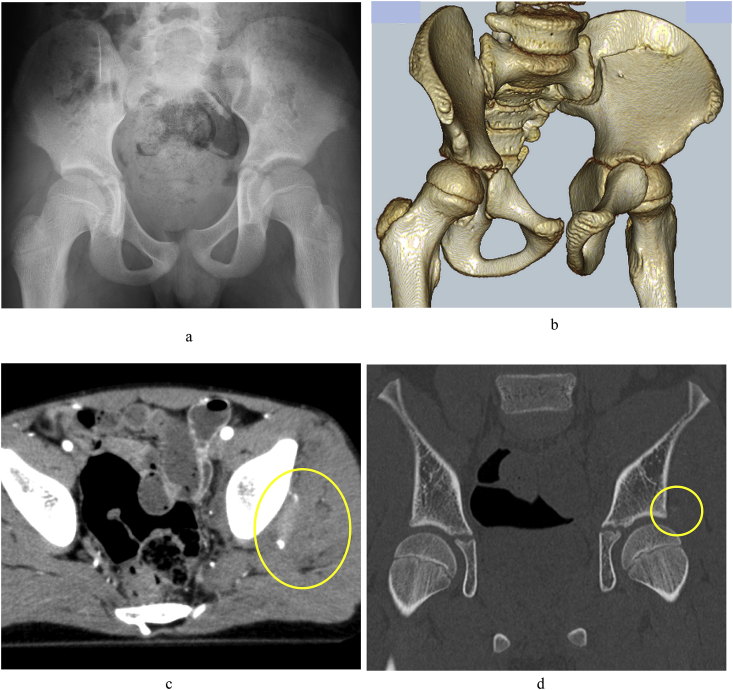
a) X-ray at the time of injury. Pubic symphysis diastasis can be seen. b) Three-dimensional CT showing left sacroiliac joint widening. Left iliac wing fracture can be seen. c) CT horizontal section. Small bone fragments and hematoma can be seen in the lateral side of the left acetabulum. d) CT coronal section showing small bone fragments on the lateral side of the left acetabulum.

We report the case of a 10-year-old boy who presented with the chief complaint of limping. His medical and family histories did not reveal any particular concerns. The patient was injured when struck by a passenger car while walking. He was transported by ambulance to the trauma center. X-ray was performed immediately after injury and revealed pubic symphysis diastasis (Fig. 1a). CT revealed sacroiliac joint widening, with left iliac wing fracture (Fig. 1b), as well as small bone fragments and hematoma in the posterior acetabular wall (Fig. 1c and d). Load relief was implemented for 4 weeks, after which load bearing was commenced. At 7 months after injury, the patient was referred to our department with the chief complaint of limping.

Physical findings: The initial examination revealed that there was no pain in the left hip. The hip ROM was as follows: flexion 140°/90°, extension 20°/−40°, abduction 30°/10°, adduction 20°/20°, external rotation 70°/60°, and internal rotation 60°/20°. The ROM was markedly limited for extension, abduction, and internal rotation. The Thomas test results were positive, and flexion contracture of the hip was observed.

**Fig. 2:**
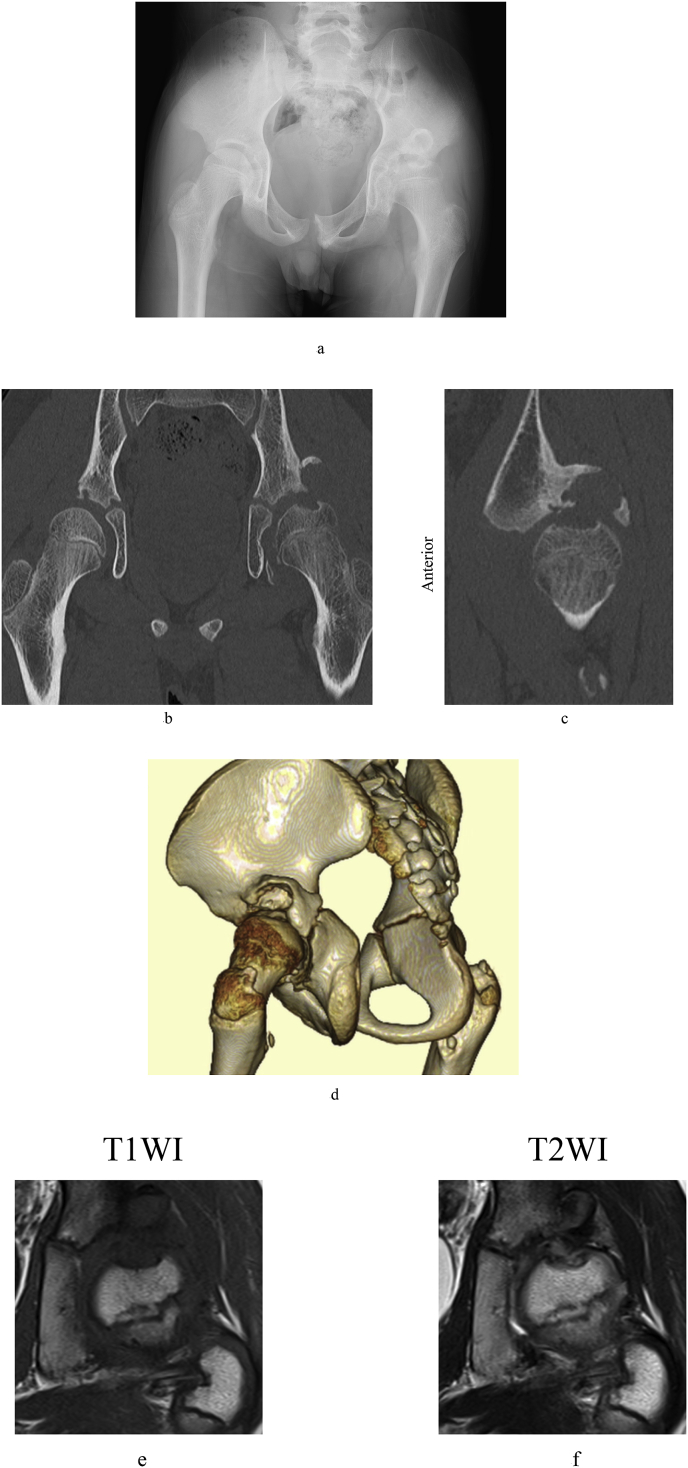
a) X-ray at 7 months after injury. Narrowing of the left hip joint space, deformity of the femoral head, narrowing of the obturator foramen, and the break in the Shenton line can be seen. b) CT coronal section at 7 months after injury. Bone fragments can be seen on the lateral side of acetabulum. A localized defect in the upper portion of the femoral head can also be seen. c) CT sagittal section showing that the posterior acetabular wall has displaced proximally. d) Three-dimensional CT showing that the posterior acetabular wall has displaced proximally. e) Low signal intensity is seen on T1-weighted MRI. f) T2-weighted MRI showing isointensity, with necrosis of the femoral head.

Imaging Findings: X-ray revealed a narrowing of the left hip joint space and deformity of the femoral head (Fig. 2a). Compared with the X-ray taken at the time of injury (Fig. 1a), the obturator foramen had narrowed, and pelvic anteversion was observed. The Shenton line was broken, and subluxation of the hip was suspected. CT showed bone fragments in the lateral wall of the acetabulum. There was a localized defect in the upper portion of the femoral head (Fig. 2b). On the sagittal section, the posterior wall of the pelvis was observed to have been dislocated proximally (Fig. 2c). Bone fragments in the posterior wall were similarly observed on three-dimensional CT (Fig. 2d). T1-weighted MRI revealed hypointensity (Fig. 2e), whereas T2-weighted imaging revealed isointensity (Fig. 2f). Femoral head necrosis was diagnosed based on imaging findings.

**Fig. 3:**
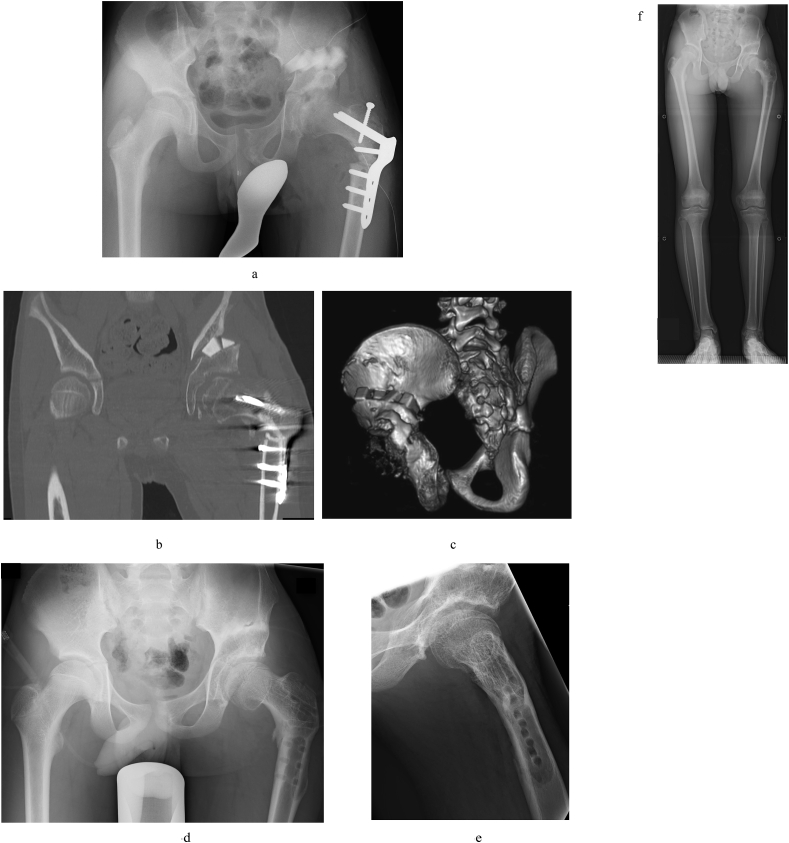
a) X-ray taken immediately after surgery. b) Postoperative CT coronal section. The femoral head has reduced, and acetabular roof coverage is good. c) Postoperative three-dimensional CT showing that artificial bone has been inserted into the osteotomy site. d) and e) X-ray taken at 1 year after surgery. f) Full-length X-ray of the bilateral legs at 2 years after surgery. A difference between the length of the legs persists.

Operative findings: Operation was performed with the patient in the lateral decubitus position. The adductor longus and gracilis muscle were completely cut using a medial approach. Next, a 15-cm incision was made from the anterior superior iliac spine through the greater trochanter along the femoral axis. The fascia lata was resected to an area of 10 × 10-cm, the sartorius muscle and rectus femoris muscle were flipped distally at the respective attachment sites, and the iliopsoas tendon was dissected. Dissection of the soft tissue alone enabled extension of the hip. Twenty degrees varus, 20° extension and 20° internal rotation femoral osteotomy and greater trochanteric epiphysiodesis were performed. IPO was performed for the pelvis. A chisel was inserted from the proximal side of the posterior wall bone fragment that had been displaced proximally, and osteotomy was performed (Fig. 3a, b, 3c). The operation time was 3 h and 35 min, and the total blood loss was 366 ml. A hip spica cast was fixed for 4 weeks after operation. The operation was performed by the senior author (SM), who has over 20 years of experience.

Patient Outcome: The treatment after operation included cast fixation for 4 weeks. Beginning at 6 weeks after surgery, partial weight-bearing was commenced. Immediately after operation, complete paralysis of the sciatic nerve was observed. Tibial nerve paralysis had recovered 2 days after surgery. Peroneal nerve paralysis improved gradually and was completely recovered by 1 year after surgery. The hip ROM at 1 year after surgery was as follows: flexion 140°/120°, extension 20°/−20°, abduction 30°/20°, adduction 20°/20°, external rotation 70°/40°, and internal rotation 60°/30°. A marked improvement in limited hip ROM was observed.

X-ray performed at 1 year after operation revealed good reduction of the femoral head and that the deformity of the femoral head had improved (Fig. 3d and e). At 2 years after operation, a difference of 21 mm in leg length persisted (Fig. 3f).

## 3 Discussion

Traumatic hip dislocation is said to occur in 0.8/100,000 of children aged <16 years per year [1], making it extremely rare. Among cases of traumatic hip dislocation, instances accompanied with posterior acetabular wall fracture are even rarer, with an incidence of 17%–33% [3]. Upon reviewing the CT images obtained immediately after the injury in the present case, small bone fragments were observed in the posterior acetabular wall with hematoma (Fig. 1c and d). Therefore, it is conceivable that in the present case, hip dislocation occurred at the time of trauma, which spontaneously reduced. In children who have insufficient ossification, bone fracture cannot be adequately evaluated by X-ray or CT. However, bone fragments in the posterior wall with insufficient ossification can be clarified on MRI [2,3]. We believe that an MRI scan should have been done after our patient's general condition had settled down, considering the possibility that hip dislocation had occurred. Furthermore, Calkins et al. [5] studied acetabular fractures using CT scans and clinical examination of the joint in 90° of flexion and neutral rotation. They concluded that a 66% compromise of the posterior wall would cause hip instability. A gray zone was also identified when the posterior wall was disrupted between 45% and 66%. All disruptions of <45% were stable [5]. In the present case, it is also possible that the large cartilage fragments of the posterior wall would have been detected if an MRI scan had been performed.

One of the most serious complications of traumatic hip dislocation is femoral head necrosis [6,7]. Reduction of a dislocated hip within 6 h of injury is most important to avoid femoral head necrosis [6,7]. As it is thought that spontaneous reduction occurred immediately after injury in the present case, reduction is believed to have occurred within 6 h. Although femoral head necrosis was observed on MRI in the present case (Fig. 2e and f), we believe that the femoral head necrosis was not attributed to the traumatic hip dislocation, but rather changes caused by friction between the femoral head and acetabular roof associated with hip subluxation. Evidence of this is that after reduction of hip subluxation, the bone head deformity improved rapidly and had completely disappeared at 1 year after surgery (Fig. 3d and e).

With regard to the operation methods, we believe that it is important to perform thorough soft tissue dissection for limited ROM of the hip [8]. Adequate hip ROM can be obtained by soft tissue dissection alone. We treated hip subluxation by means of femoral head osteotomy. The femoral head subluxation was reduced by femoral head osteotomy, and dissection of the joint capsule was not required. The posterior instability of the hip caused by a defect in the posterior acetabular wall was treated by IPO. IPO involves pelvic osteotomy developed for hip dislocation in children with strong defects of the posterior acetabular wall, and it enables proper acetabular roof coverage of the lateral and posterior sides [9]. However, at 2 years after surgery, a difference of 21 mm in the length of the legs persisted (Fig. 3f). In the future, valgus femoral osteotomy and/or leg lengthening might be needed.

In the present case, complete paralysis of the sciatic nerve developed after operation. Sierra et al. [10] reported that among 1760 patients who underwent periacetabular osteotomy, 36 (2.1%) developed sciatic nerve paralysis and femoral nerve paralysis. Among these patients, 17 (47.2%) recovered completely, and the median period until recovery or symptom stabilization was 5.5 months. Although they cannot clarify which patients were at high risk of nerve injury, femoral nerve injury has good prognosis, and recovery of sciatic nerve injury is believed to depend on the cause and severity of injury. Sierra et al. also stated that they believed that exploration may be warranted if direct nerve trauma is suspected [10]. In the present case, the tibial nerve paralysis improved quickly, and the peroneal nerve paralysis recovered slowly. We believe that exploration would be needed if recovery of paralysis is poor. As the cause of sciatic nerve paralysis, we believe that bleeding occurring at the time of traumatic hip dislocation caused the sciatic nerve to adhere to the pelvis. During operation, insertion of the Salter's retoractor into the sciatic notch may have caused damage.

With regard to conservative treatment of sciatic nerve paralysis, if immediately after surgery, to reduce nerve stretching, the hip is extended and the knee is flexed [11]. This present case was not able to be done this position because he was in a cast. Also, as sciatic nerve paralysis is slow to recover, physical therapy to maintain ROM, bracing during ambulation and night time splinting are necessary to prevent contracture of the Achilles tendon [11].

In the present case, hip subluxation was noticed at 7 months after injury. We believe that the subluxation progressed gradually after commencing weight-bearing at 4 weeks after injury. As expected, the findings of physical examination are important, and when limitation to hip extension, abduction, and internal rotation is observed, hip subluxation should be suspected. At such time, if a break in the Shenton line is observed on X-ray, femoral head subluxation is certain [12], and additional examination by CT and MRI is important for reaching a definitive diagnosis.

## 4 Conclusion

In pediatric pelvic fractures, MRI should be taken for assessing cartilage components injury.

It is important to perform thorough soft tissue dissection for limited ROM of the hip. Adequate hip ROM can be obtained by soft tissue dissection alone.

If limitation to hip extension, abduction, and internal rotation is observed with a break in the Shenton line on X-ray findings, subluxation of the hip must be suspected.

## Ethical approval

All procedures performed in this study were in accordance with ethical standards of the Ethics Committee of Nagasaki University Graduate School of Biomedical Sciences (approval number:20061525)

## Sources of funding

None.

## Author contribution

Shohei Matsubayashi, MD., PhD: Corresponding Author.

Eri Kanzaki, MD: data collection.

Ritsu Tsujimoto, MD., PhD: data analysis.

Makoto Osaki, MD., PhD: interpretation.

Akifusa Wada, MD., PhD: study concept.

## Registration of research studies

Name of the registry: Neglected subluxation of the hip after trauma: a case report.

Unique Identifying number or registration ID: researchregistry5737.

Hyperlink to your specific registration (must be publicly accessible and will be checked): none.

## Guarantor

Shohei Matsubayashi.

## Provenance and peer review

Not commissioned, externally peer reviewed.

## Declaration of competing interest

None.
