# Supplementary material for: Neglected subluxation of the hip after trauma: A case report
Source: Ann Med Surg (Lond). 2020 Jul 22;57:223–7. doi: 10.1016/j.amsu.2020.07.035 (PMC7415628; doi:10.1016/j.amsu.2020.07.035)
Supplement: Multimedia component 1 [file mmc1.docx]

| **SCARE 2018 Checklist** | | | |
| --- | --- | --- | --- |
| **Topic** | **Item** | **Checklist item description** | **Page Number** |
| **Title** | **1** | The words “case report” should appear in the title. The title should also describe the area of focus (e.g. presentation, diagnosis, surgical technique or device or outcome). | 1 |
| **Key Words** | **2** | 3 to 6 key words that identify areas covered in this case report (include "case report" as one of the keywords). | 2 |
| **Abstract** | **3a** | Introduction — Describe what is unique or educational about the case (i.e. what does this work add to the surgical literature, and why is this important?). | 1  1  1-2  2 |
|  | **3b** | Presenting complaint and investigations – describe the patient's main concerns and important clinical findings. |  |
|  | **3c** | The main diagnoses, therapeutics interventions, and outcomes. |  |
|  | **3d** | Conclusion — Describe the main lessons to “take-away” from this case study |  |
| **Introduction** | **4** | Background – summarise what is unique or educational about the case. Give reference to the relevant surgical literature and current standard of care. The background should be referenced, and 1-2 paragraphs in length. | 3 |
| **Patient Information** | **5a** | Demographic details – include de-identified demographic details on patient age, sex, ethnicity, occupation. Where possible, include other useful pertinent information e.g. body mass index and hand dominance. | 3  3-4  3-4  3 |
|  | **5b** | Presentation - describe the patient’s presenting complaint (symptoms). Describe the patient’s mode of presentation (brought in by ambulance or walked into Emergency room or referred by family physician). |  |
|  | **5c** | Past medical and surgical history, and relevant outcomes from interventions |  |
|  | **5d** | Other histories – Describe the patient’s pharmacological history including allergies, psychosocial history (Drug, smoking, and if relevant, accommodation, walking aids), family history including relevant genetic information. |  |
| **Clinical Findings** | **6** | Describe the relevant physical examination and other significant clinical findings. Include clinical photographs where relevant and where consent has been given. | 4 |
| **Timeline** | **7** | Inclusion of data which allows readers to establish the sequence and order of events in the patient's history and presentation (using a table or figure if this helps). Delay from presentation to intervention should be reported. | 3-4 |
| **Diagnostic Assessment** | **8a** | Diagnostic methods – describe all investigations taken to arrive at methods: physical exam, laboratory testing, radiological imaging, histopathology. | 4-5  4-5  4-5  4-5 |
|  | **8b** | Diagnostic challenges – describe what was challenging about the diagnoses, where applicable, for example access, financial, cultural. |  |
|  | **8c** | Diagnostic reasoning – Describe the differential diagnoses and why they were considered. |  |
|  | **8d** | Prognostic characteristics when applicable (e.g. tumour staging or for certain genetic conditions). Include relevant radiological or histopathological images in this section. |  |
| **Therapeutic Intervention** | **9a** | Pre-intervention considerations – if there were patient-specific optimisation measures taken prior to surgery or other intervention these should be included e.g. treating hypothermia/hypovolaemia/hypotension in a burns patient, Intensive care unit treatment for sepsis, dealing with anticoagulation/other medications, etc. | 5  5  5  5 |
|  | **9b** | Interventions – describe the type(s) of intervention(s) deployed (pharmacologic, surgical, physiotherapy, psychological, preventive). Describe the reasoning behind this treatment offered. Describe any concurrent treatments (antibiotics, analgesia, anti-emetics, nil by mouth, Venous thrombo-embolism prophylaxis, etc). Medical devices should have manufacturer and model specifically mentioned. |  |
|  | **9c** | Intervention details – describe what was done and how. For surgery include details on; anaesthesia, patient position, use of tourniquet and other relevant equipment, prep used, sutures, devices, surgical stage (1 or 2 stage, etc). For pharmacological therapies include information on the formulation, dosage, strength, route, duration, etc. Include intra-operative photographs and/or video or relevant histopathology in this section. Degree of novelty for a surgical technique/device should be mentioned e.g. "first in human". |  |
|  | **9d** | Who performed the procedure - operator experience (position on the learning curve for the technique if established, specialisation and prior relevant training). For example, “junior resident with 3 years of specialised training” |  |
|  | **9e** | Changes – if there were any changes in the interventions, describe these details with the rationale. |  |
| **Follow-up and**  **Outcomes** | **10a** | Follow-up – describe 1) When the patients was followed up. 2) Where. 3) How (imaging, tests, scans, clinical examination, phone call), and 4) whether there were any specific post-operative instructions. Future surveillance requirements - e.g. imaging surveillance of endovascular aneurysm repair or clinical exam/ultrasound of regional lymph nodes for skin cancer. | 6  6  6 |
|  | **10b** | Outcomes - Clinician assessed and (when appropriate) patient-reported outcomes (e.g. questionnaire details). Relevant photographs/radiological images should be provided e.g. 12 month follow-up. |  |
|  | **10c** | Intervention adherence/compliance - where relevant how well patient adhered to and tolerated their treatment. For example, post-operative advice (heavy lifting for abdominal surgery) or tolerance of chemotherapy and pharmacological agents |  |
|  | **10d** | Complications and adverse events – all complications and adverse or unanticipated events should be described in detail and ideally categorised in accordance with the Clavien-Dindo Classification. How they were prevented, diagnosed and managed. Blood loss, operative time, wound complications, re-exploration/revision surgery, 30-day post-op and long-term morbidity/mortality may need to be specified. If there were no complications or adverse outcomes this should also be included. |  |
| **Discussion** | **11a** | Strengths – describes the strengths of this case | 7  9  6-10  9-10  10 |
|  | **11b** | Weaknesses and limitations in your approach to this case. For new techniques or implants - contraindications and alternatives, potential risks and possible complications if applied to a larger population. If relevant, has the case been reported to the relevant national agency or pharmaceutical company (e.g. an adverse reaction to a device) |  |
|  | **11c** | Discussion of the relevant literature, implications for clinical practice guidelines and any relevant hypothesis generation. |  |
|  | **11d** | The rationale for your conclusions. |  |
|  | **11e** | The primary “take-away” lessons from this case report. |  |
| **Patient Perspective** | **12** | When appropriate the patient should share their perspective on the treatments they received. |  |
| **Informed Consent** | **13** | Did the patient give informed consent for publication? Please provide if requested by the journal/editor. If not given by the patient, explain why e.g. death of patient and consent provided by next of kin or if patient/family untraceable then document efforts to trace them and who within the hospital is acting as a guarantor of the case report. | 3 |
| **Additional Information** | **14** | Conflicts of Interest, sources of funding, institutional review board or ethical committee approval where required. | 3 |
